# A Novel Instantaneous Phase Detection Approach and Its Application in SSVEP-Based Brain-Computer Interfaces

**DOI:** 10.3390/s18124334

**Published:** 2018-12-07

**Authors:** Xiangdong Huang, Jingwen Xu, Zheng Wang

**Affiliations:** 1 School of Electrical and Information Engineering, Tianjin University, Tianjin 300072, China; xdhuang@tju.edu.cn (X.H.); xujingwen18@163.com (J.X.); 2 College of Intelligence and Computing, Tianjin University, Tianjin 300072, China

**Keywords:** fully-traversed DFT, phase estimator, direct phase extraction, spectral leakage, SSVEP

## Abstract

This paper proposes a novel phase estimator based on fully-traversed Discrete Fourier Transform (DFT) which takes all possible truncated DFT spectra into account such that it possesses two merits of ‘direct phase extraction’ (namely accurate instantaneous phase information can be extracted without any correction) and suppressing spectral leakage. This paper also proves that the proposed phase estimator complies with the 2-parameter joint estimation model rather than the conventional 3-parameter joint model. Numerical results verify the above two merits and demonstrate that the proposed estimator can extract phase information from noisy multi-tone signals. Finally, real data analysis shows that fully-traversed DFT can achieve a better classification on the phase of steady-state visual evoked potential (SSVEP) brain-computer interface (BCI) than the conventional DFT estimator does. Besides, the proposed phase estimator imposes no restrictions on the relationship between the sampling rates and the stimulus frequencies, thus it is capable of wider applications in phase-coded SSVEP BCIs, when compared with the existing estimators.

## 1. Introduction

Estimating the frequency, the phase and the amplitude of a signal is a standard classical problem of signal processing. In particular, phase estimation has been widely applied in synchronization in communication [1], power analysis [2], speech enhancement [3], GPS navigation [4] and much more. Until now, phase estimators based on direct Discrete Fourier Transform (DFT) are the mainstream [5].

In [5], Liguori pointed out that, the existing DFT-based phase estimators heavily rely on the result of frequency estimate. To illustrate this dependency, let us study the sampled version (the sampling rate is $F_{s}$) of a complex exponential signal $x\left(t\right)=a_{0}$exp$\left[j\left(2\pi f_{0}t+\theta_{0}\right)\right]$ as

(1) $$x\left(n\right)=x\left(t\right)\left|{}_{t=n\Delta t}=a_{0}e^{j(2\pi f_{0}n\Delta t+\theta_{0})},\;n=0,\ldots ,N-1,\right.$$

where $a_{0}$, $f_{0}$, $\theta_{0}$ are amplitude, frequency and phase respectively and Δ*t* is the sampling interval 1/$F_{s}$. Accordingly, the frequency resolution of the *N*-point DFT X(k) is Δ*f* = $F_{s}$/*N* = 1/*N*Δ*t*. Assume the peak DFT bin is at *k* = $k_{0}$, $k_{0}$ ∈ $Z^{+}$ ($Z^{+}$ refers to the set of positive integer numbers). Then, it can be deduced that the ideal phase value of $X(k_{0})$ is

(2) $$\theta_{0}=\arg\left(X(k_{0})\right)-\pi \delta \left(N-1\right)/N.$$

The variable δ in (2) is the fractional frequency offset (−0.5≤δ<0.5) with the value $\delta =f_{0}/\Delta f-k_{0}$, which is closely related to two sampling cases (coherent sampling or noncoherent sampling). For the coherent sampling case, i.e., $f_{0}=k_{0}\Delta f$, δ=0 and thus the sequence {x(n),n=0,1,⋯, *N* − 1} exactly covers $k_{0}$ signal periods, it can be easily inferred from (2) that the phase $\theta_{0}$ can be estimated by directly taking the phase at the peak bin. Nevertheless, in case of noncoherent sampling, i.e., $f_{0}=\left(k_{0}+\delta \right)\Delta f$, δ≠0 and thus the sequence does not exactly contain integer times of signal periods, it can be inferred from (2) that the phase estimate $\theta_{0}$ is related to both the peak index $k_{0}$ and the frequency offset δ.

Up to now, a lot of frequency estimators have been proposed to estimate the fractional offset δ. For example, Offelli proposed an energy-based approach [6] in which the frequency offset of some component can be derived from the energy summation of the windowed DFT spectral bins around the peak bin. Other approaches based on interpolated FFT were reported in [2,7,8,9], in which δ is calculated via interpolation between several successive high-amplitude DFT bins centered with peak bin. In recent years, a lot of high-accuracy interpolation-based estimators such as Provencher estimator [10], Jacobsen estimator [11], Candan estimator [12], and phase difference-based estimator [13] were proposed. However, in [5,14,15], Liguori emphasized that “the bias of frequency estimate will introduce the uncertainty propagation to the phase acquisition”. In other words, the error of frequency offset δ in (2) will inevitably give rise to the error of phase estimate $\theta_{0}$ accordingly.

Besides, spectral leakage is also a non-ignorable factor of degrading DFT-based phase estimators. In essence, for the non-coherent sampling case, performance degradation of phase estimation actually results from DFT’s inherent spectral leakage effect [16]. Therefore, to enhance the accuracy of phase estimation, some approach capable of suppressing spectral leakage is expected to be developed.

This paper proposes a novel approach of correcting DFT spectra which can alleviate phase estimate’s dependence on frequency estimate (or rather, the dependence on frequency offset δ). This improvement actually attributes to the property that the proposed fully-traversed DFT spectrum has a much slighter spectral leakage compared to original uncorrected DFT spectrum in non-coherent sampling case. To verify these advantages, we also apply this corrected DFT-based phase estimator into a phase-coded steady-state visual evoked potential-based brain-computer interface (SSVEP-based BCI).

Recently, for the purpose of increasing realizable targets, phase information is popularly integrated into frequency-coding in SSVEP-BCI, such as joint/mixed frequency and phase coding [17,18,19]. In [20], Lee introduced one scheme of phase coding in SSVEP-BCI system, in which 8 LEDs flickering at 31.25 Hz with the phase interval $45^{\circ }$ were used to represent 8 cursor functions. However, the phase decoding in [20] was realized through detecting the maximum amplitude peak of the averaged SSVEP waveform, i.e., it was realized in time domain instead of frequency domain. In [21], Lee segmented one stimulus into reference epoch and phase-shift epoch, in which the phase differences between these two epochs were used to distinguish different targets. Shortly after that, in [22], Lee designed six SDFS (stepping delay flickering sequences) with stimulus frequency of 32 Hz, among which 6 different phase-tagged objects are represented by 6 distinct delay segments such that they can be detected by the normalized power of averaged responses. However, SSVEP-BCIs in [21,22] actually employ special stimulus sequences to facilitate phase decoding, which may be not suitable for the common situations of phase measurement. In [23], to increase the number of visual stimuli, Gao and Jia proposed a frequency and phase mixed coding scheme using multiple frequencies. Then, Gao proposed the phase constrained canonical correlation analysis (p-CCA) to better distinguish frequency-tagged stimuli from multichannel SSVEPs [24], in which the phase information was estimated from the FFT result of the SSVEP records over the occipital cortex. Following this, Gao et.al introduced phase coding in CCA to discriminate six $60^{\circ }$-interval targets [25]. However, the stimuli frequencies in [23,24,25] were actually deliberately chosen such that each stimuli frequency exactly equals the integer times of FFT resolution and thus the frequency offset is also exactly zero. Therefore, in SSVEP-BCIs, novel phase estimator capable of detecting the phases of stimuli with any frequency offset is expected to be developed.

This paper will demonstrate that the proposed phase corrected DFT-based estimator is in accordance with the above demand of SSVEP-BCIs. Experimental results show that, the proposed phase estimator is superior to the conventional estimator in SSVEP phase extraction.

The rest of this paper is organized as follows. Section 2 introduces the derivation of the proposed fully-traversed DFT. Section 3 elaborates the properties of the proposed fully-traversed DFT in the noiseless case. Section 4 gives accuracy analysis of this phase estimator in noisy case. Section 5 demonstrates the proposed DFT-based phase estimator’s superiority to conventional phase estimators in the phase-coded SSVEP-BCI with auto-calibration. Finally, we conclude with a summary of results in Section 6.

## 2. Derivation of the Fully-Traversed DFT Spectrum

### 2.1. Phase Property of DFT Spectrum

Consider a sampled signal {x(0),x(1),⋯,x(*N* − 1)} (or denoted as the vector $x_{0}$). As is known, the normalized conventional DFT of x(n) is

(3) $$X\left(k\right)=\frac{1}{N}\sum_{n=0}^{N-1}x\left(n\right)e^{-j\frac{2\pi }{N}kn},\;k=0,1,\ldots ,N-1.$$

Without loss of generality, assume that Δω=2π/N and $\omega_{0}=\beta \Delta \omega =\left(k_{0}+\delta \right)\Delta \omega$, $k_{0}\in Z^{+}$ and −0.5≤δ<0.5. Substituting the complex exponential sequence x(n)= exp(j$\left(\omega_{0}n+\theta_{0})\right)$ into (3) yields

(4) $$\begin{array}{cc} X(k) & =\frac{1}{N}\sum_{n=0}^{N-1}e^{j\left(\frac{2\pi }{N}\beta n+\theta_{0}\right)}e^{-j\frac{2\pi }{N}kn}\\ & =\frac{e^{j\theta_{0}}}{N}\sum_{n=0}^{N-1}e^{j\frac{2\pi }{N}\left(\beta -k\right)n}\\ & =\frac{e^{j\theta_{0}}}{N}\sum_{n=0}^{N-1}e^{j\frac{2\pi }{N}\left(k_{0}-k+\delta \right)n}. \end{array}$$

Using geometric series summation and Euler equation, one can further deduce (4) as

(5) $$X\left(k\right)=\left\{\begin{array}{c} e^{j\left[\theta_{0}+\frac{N-1}{N}\left(k_{0}+\delta -k\right)\pi \right]}\cdot \frac{\sin\left[(k_{0}+\delta -k)\pi \right]}{N\sin\left[(k_{0}+\delta -k)\pi /N\right]},\delta \neq 0\\ e^{j\theta_{0}},\delta =0 \end{array}\right.$$

Substituting the peak index $k=k_{0}$ into (4) and synthesize the two cases of δ≠0 and δ=0, we can derive a uniform expression of the phase value at peak DFT bin as

(6) $$\phi_{X}\left(k_{0}\right)=\theta_{0}+\left(N-1\right)/N\cdot \delta \pi$$

Obviously, (6) is in accordance with (1).

From (6), it can be found that, only for the coherent sampling case (the frequency offset δ=0), the observed phase $\phi_{X}\left(k_{0}\right)$ at the peak DFT bin is an accurate estimate. In contrast to this, for the non-coherent sampling case (δ≠0), in order to estimate the phase, δ has to be estimated in advance. As a result, the phase estimator based on conventional DFT heavily depends on the frequency offset δ.

### 2.2. The Proposed Fully-Traversed DFT Spectrum

In this work, we aim to develop a phase estimator capable of removing the dependency on the frequency offset δ. To be specific, our goal is to construct a corrected DFT spectrum whose peak bin provides accurate phase information whether δ=0 or not.

To derive the proposed fully-traversed DFT, it is necessary to study the relationship between the ideal Fourier transform and the conventional DFT. As known to us, the ideal Fourier transform X(jω) of an infinite-length sequence {x(n)}={⋯,x(−*N* + 1),⋯,x(0),⋯,x(*N* − 1),⋯} is

(7) $$X\left(j\omega \right)=\sum_{n=-\infty }^{+\infty }x\left(n\right)e^{-jn\omega }$$

However, X(jω) cannot be realized, since the calculation in (7) consumes innumerous samples and memory. Thus, in practical applications, X(jω) is replaced by the conventional normalized DFT X(k) defined in (3). Furthermore, if we define a sequence $x_{0}\left(n\right)$, which is truncated from the infinite-length sequence {x(n),−∞≤n≤∞}, i.e.,

(8) $$\begin{array}{c} x_{0}\left(n\right)=\left\{\begin{array}{c} x(n),\;n\in [0,N-1]\\ 0,\;\;\;\mathrm{others} \end{array},\right. \end{array}$$

then, apparently, combining (3) with (7), (8) yields a representation of X(k) as

(9) $$\begin{array}{cc} X(k) & =\frac{1}{N}X_{0}\left(j\omega \right)\left|{}_{\omega =k2\pi /N}\right.\\ & =\frac{1}{N}\sum_{n=-\infty }^{+\infty }x_{0}\left(n\right)e^{-jnk2\pi /N}\\ & =\frac{1}{N}\sum_{n=0}^{N-1}x\left(n\right)e^{-jnk2\pi /N} \end{array}$$

Now, let us focus on the sample x(0) at which the ideal phase $\theta_{0}$ is located. It can be inferred from (9) that conventional DFT only considers one truncation case. However, there are *N* truncated sequences $x_{m}$ (m=0,1,…,N−1) containing x(0) listed as

(10) $$\begin{array}{c} \begin{array}{c} x_{0}=\left\{x\left(0\right),x\left(1\right),\ldots ,x\left(N-2\right),x\left(N-1\right)\right\},\\ x_{1}=\left\{x\left(-1\right),x\left(0\right),\ldots ,x\left(N-3\right),x\left(N-2\right)\right\},\\ \;\;\;\;\;\;\;\;\;\;\vdots \\ x_{N-1}=\left\{x\left(-N+1\right),x\left(-N+2\right),\ldots ,x\left(-1\right),x\left(0\right)\right\}, \end{array} \end{array}$$

where the elements of sequence $x_{m}=\{x_{m}\left(n\right),n=0,\cdots ,$*N* − 1} are

(11) $$\begin{array}{c} x_{m}\left(n\right)=\left\{\begin{array}{c} x(n-m),n\in [0,N-1]\\ 0,\;\;\;\;\;\;\;\mathrm{others} \end{array}\right. \end{array}$$

Similar to the discrete spectrum of the truncated sequence $x_{0}$ expressed in (8), a reasonable discrete spectrum for $x_{m}=\{x\left(-m\right),x($−*m* + 1),⋯,x(−m + *N* − 1)} should be

(12) $$\begin{array}{cc} X_{m}\left(k\right) & =\frac{1}{N}\sum_{n=-m}^{N-1-m}x\left(n\right)e^{-jnk2\pi /N},\\ & \;m,k=0,1,\ldots ,N-1. \end{array}$$

Furthermore, aiming to rewrite $X_{m}\left(k\right)$ in the form of conventional DFT in which variable *n* ranges from 0 to *N* − 1, we should detach the series summation in (12) into two terms as

(13) $$\begin{array}{c} X_{m}\left(k\right)=\frac{1}{N}\sum_{n=-m}^{-1}x\left(n\right)e^{-jnk2\pi /N}\\ +\frac{1}{N}\sum_{n=0}^{N-m+1}x\left(n\right)e^{-jnk2\pi /N}, \end{array}$$

The first summation in (13) can be denoted as

(14) $$\begin{array}{c} \frac{1}{N}\sum_{n=-m}^{-1}x\left(n\right)e^{-jnk2\pi /N}\\ \underline{\underline{n^{\prime }=n+N}}\;\;\frac{1}{N}\sum_{n^{\prime }=N-m}^{N-1}x\left(n^{\prime }-N\right)e^{-j\left(n^{\prime }-N\right)k2\pi /N}\\ =\frac{1}{N}\sum_{n=N-m}^{N-1}x\left(n-N\right)e^{-jnk2\pi /N}. \end{array}$$

Hence, it is necessary to introduce *N* new truncated sequences ${\tilde{x}}_{m}$ (m=0,1,…,N−1) whose elements are

(15) $$\begin{array}{cc} {\tilde{x}}_{m}\left(n\right) & =\left\{\begin{array}{c} x(n)\;\;\;\;\;\;\;\;\;,n=0,\ldots ,N-m-1\\ x(n-N),n=N-m,\ldots ,N-1 \end{array}.\right. \end{array}$$

In terms of (15), *N* sequences ${\tilde{x}}_{0}∼{\tilde{x}}_{N-1}$ are listed as

(16) $$\begin{array}{c} \begin{array}{c} {\tilde{x}}_{0}=\{x\left(0\right),x\left(1\right),\ldots ,x\left(N-2\right),x\left(N-1\right)\},\\ {\tilde{x}}_{1}=\{x\left(0\right),x\left(1\right),\ldots ,x\left(N-2\right),x\left(-1\right)\},\\ \;\;\;\;\;\;\;\;\;\;\vdots \\ {\tilde{x}}_{N-1}=\left\{x\left(0\right),x\left(-N+1\right),\ldots ,x\left(-2\right),x\left(-1\right)\right\}. \end{array} \end{array}$$

Then, combining (13) with (15), we have

(17) $$\begin{array}{c} X_{m}\left(k\right)=\frac{1}{N}\sum_{n=0}^{N-1}{\tilde{x}}_{m}\left(n\right)e^{-jnk2\pi /N}=DFT\left[{\tilde{x}}_{m}\left(n\right)\right],\\ \;m=0,1,\ldots ,N-1. \end{array}$$

Finally, averaging all the DFT results of ${\tilde{x}}_{0}∼{\tilde{x}}_{N-1}$ yields the proposed corrected spectrum, i.e.,

(18) $$\begin{array}{c} Y\left(k\right)=\frac{1}{N}\sum_{m=0}^{N-1}X_{m}\left(k\right),\;\;\;k=0,1,\ldots ,N-1. \end{array}$$

From (8)–(18), it can be noticed that, the center sample x(0) fully traverses all the possible starting positions of *N* truncated sequences $x_{m}$ (m=0,1,…,N−1) in (10). Moreover, all the DFT results (17) of these traversed sub-sequences are fully averaged in (18) to yield the final spectrum Y(k) in (18). Hence, this novel spectral analysis is named as *Fully-traversed DFT*, whose dataflow is summarized as:

**step** **1.**  Construct *N* *N*-length sub sequences $x_{0}∼x_{N-1}$ from the given (2N−1) input samples x(−N+1),⋯,x(0), ⋯,x(N−1), as (10) lists;
**step** **2.**  For each index m,m=0,1,⋯,N−1, circularly left move *m* samples of $x_{m}=\{x\left(-m\right),\cdots ,$ x(0),⋯,x(−m + N − 1)} to generate *N* sequences ${\tilde{x}}_{m}=\left\{x\left(0\right),x\left(1\right),\cdots ,x\left(N-m+1\right),x\left(-m\right),\cdots ,x\left(-1\right)\right\}$.
**step** **3.**  Implement normalized DFT on each ${\tilde{x}}_{m}$ to obtain the discrete sub-spectrum $X_{m}\left(k\right),m=0,\cdots ,\;$*N* − 1;
**step** **4.**  Average all *N* sub-spectra $X_{m}\left(k\right)$ to acquire the proposed phase corrected spectrum Y(k).

## 3. Property of Phase Estimation in the Noiseless Circumstance

### 3.1. Single-Tone Case

Consider a single-tone signal x(n) = exp(j$\left(\omega_{0}n+\theta_{0})\right)$, $\omega_{0}=\beta \Delta \omega =\left(k_{0}+\delta \right)2\pi /N$. Since Y(k) is obtained by averaging $X_{0}\left(k\right)∼X_{N-1}\left(k\right)$, substituting x(n) into $X_{m}\left(k\right)$ in (12) and (18) yields

(19) $$\begin{array}{cc} Y(k) & =\frac{1}{N}\sum_{m=0}^{N-1}\left[\frac{1}{N}\sum_{n=-m}^{-m+N-1}e^{j\left(\beta 2n\pi /N+\theta_{0}\right)}e^{-jnk2\pi /N}\right]\;\;\;\;\\ & \underline{\underline{n^{\prime }=n+m}}\frac{\;e^{j\theta_{0}}}{N^{2}}\sum_{m=0}^{N-1}\left[\sum_{n^{\prime }=0}^{N-1}e^{j\left(\beta -k\right)2\left(n^{\prime }-m\right)\pi /N}\right]\\ & =\frac{\;e^{j\theta_{0}}}{N^{2}}\sum_{m=0}^{N-1}e^{-j(\beta -k)2m\pi /N}\left[\sum_{n=0}^{N-1}e^{j(\beta -k)2n\pi /N}\right] \end{array}$$

Obviously, (19) can be rewritten as

(20) $$\begin{array}{cc} Y\left(k\right)\;=e^{j\theta_{0}} & \left(\frac{e^{j\theta_{0}}}{N}\sum_{m=0}^{N-1}e^{-j\frac{2\pi }{N}\left(k_{0}-k+\delta \right)m}\right)\cdot \\ & {\left(\frac{e^{j\theta_{0}}}{N}\sum_{n=0}^{N-1}e^{-j\frac{2\pi }{N}\left(k_{0}-k+\delta \right)n}\right)}^{*} \end{array}$$

where the superscript ‘∗’ represents complex conjugate operation. Then, combining (20) with (4) yields

(21) $$\begin{array}{c} Y\left(k\right)\;=e^{j\theta_{0}}X\left(k\right)X^{*}\left(k\right)=e^{j\theta_{0}}{\left|X(k)\right|}^{2},\;\\ k=0,1,\ldots ,N-1. \end{array}$$

Taking the phase part of (21), we can obtain the phase spectrum $\varphi_{Y}\left(k\right)$ as

(22) $$\begin{array}{c} \varphi_{Y}\left(k\right)=\theta_{0},\;k=0,1,\ldots ,N-1. \end{array}$$

Equation (22) shows that, for the signal x(n) = exp$\left(j\left(\omega_{0}n+\theta_{0}\right)\right)$, −N+1≤n≤N−1, the phase values at all *N* spectral bins (including the peak bin $k=k_{0}$) uniformly equal the ideal phase $\theta_{0}$ (also refers to the instantaneous phase of the center sample x(0)). In other words, the synthesized spectrum Y(k) directly provides the accurate phase estimate at any spectral bin, thus removing the conventional DFT-based phase estimator’s dependency on the frequency offset δ.

Furthermore, since Y(k) is obtained by linearly averaging the *N* sub DFT spectra as (18) shows, the proposed fully-traversed DFT spectrum is of linearity, also. Thus, for a single-tone signal with amplitude $a_{0}\;\left(a_{0}\neq 1\right)$, the following holds

(23) $$\begin{array}{c} Y(k)=a_{0}e^{j\theta_{0}}\frac{\sin^{2}\left[(k_{0}+\delta -k)\pi \right]}{N^{2}\sin^{2}\left[(k_{0}+\delta -k)\pi /N\right]},\;k=0,1,\ldots ,N-1, \end{array}$$

among which the peak spectral bin is

(24) $$\begin{array}{c} Y(k_{0})=a_{0}e^{j\theta_{0}}\frac{\sin^{2}\left(\delta \pi \right)}{N^{2}\sin^{2}\left(\delta \pi /N\right)}. \end{array}$$

### 3.2. Multi-Tone Case

Consider a signal containing *Q* (Q≥2) components expressed as

(25) $$x\left(n\right)=\sum_{q=1}^{Q}a_{q}e^{j(\omega_{q}n+\theta_{q})}=\sum_{q=1}^{Q}a_{q}e^{j\left[\left(k_{q}+\delta_{q}\right)\Delta \omega n+\theta_{q}\right]},\;k_{q}\in Z^{+},0<\left|\delta_{q}\right|<0.5.$$

Since the conventional DFT is a linear transform, combining (5) and (25), its multi-tone spectrum X(k) is composed of *Q* single-tone spectra, i.e.,

(26) $$\begin{array}{c} \begin{array}{cc} X\left(k\right) & =\sum_{q=1}^{Q}X_{q}\left(k\right)\\ & =\sum_{q=1}^{Q}a_{q}e^{j\left[\theta_{q}+\frac{N-1}{N}\left(k_{q}+\delta_{q}-k\right)\right]}\frac{\sin\left[(k_{q}+\delta_{i}-k)\pi \right]}{N\sin\left[(k_{q}+\delta_{q}-k)\pi /N\right]},k=0,\ldots ,N-1 \end{array} \end{array}$$

Similarly, since the proposed phase corrected DFT spectrum is also of linearity, combining (5) with (23), its multi-tone spectrum Y(k) equals the summation of *Q* single-tone spectra as

(27) $$\begin{array}{c} \begin{array}{c} Y\left(k\right)=\sum_{q=1}^{Q}Y_{q}\left(k\right)\\ \;\;\;\;\;=\sum_{q=1}^{Q}a_{q}e^{j\theta_{q}}\frac{\sin^{2}\left[(k_{q}+\delta_{q}-k)\pi \right]}{N^{2}\sin^{2}\left[(k_{q}+\delta_{q}-k)\pi /N\right]},k=0,\ldots ,N-1 \end{array} \end{array}$$

Furthermore, to measure the phase of the *i*-th tone, the peak bin at $k=k_{i}$ should be focused. Therefore, the conventional DFT spectrum $X(k_{i})$ can be written as

(28) $$\begin{array}{cc} X(k_{i}) & =X_{i}\left(k_{i}\right)+\sum_{q\neq i}^{}X_{q}\left(k_{i}\right)\\ & =a_{i}e^{j(\theta_{i}+\frac{N-1}{N}\delta_{i})}\frac{\sin(\delta_{i}\pi )}{N\sin(\delta_{i}\pi /N)}+\sum_{q\neq i}^{}a_{q}e^{j\left[\theta_{q}+\frac{N-1}{N}\left(k_{q}+\delta_{q}-k_{i}\right)\right]}\frac{\sin\left[(k_{q}+\delta_{q}-k_{i})\pi \right]}{N\sin\left[(k_{q}+\delta_{q}-k_{i})\pi /N\right]}. \end{array}$$

Similarly, the proposed fully-traversed DFT spectrum $Y(k_{i})$ can be expressed as

(29) $$\begin{array}{cc} Y(k_{i}) & =Y_{i}\left(k_{i}\right)+\sum_{q\neq i}^{}Y_{q}\left(k_{i}\right)\\ & =a_{i}e^{j\theta_{i}}\frac{\sin^{2}\left(\delta_{i}\pi \right)}{N^{2}\sin^{2}\left(\delta_{i}\pi /N\right)}+\sum_{q\neq i}^{}a_{q}e^{j\theta_{q}}\frac{\sin^{2}\left[(k_{q}+\delta_{q}-k_{i})\pi \right]}{N^{2}\sin^{2}\left[(k_{q}+\delta_{q}-k_{i})\pi /N\right]}. \end{array}$$

In (29), the first term is the expected *i*-th single-tone spectrum which directly provides the phase estimate, and the second item represents the interference of other tones. Obviously, for either the conventional DFT spectrum or the fully-traversed DFT spectrum, the accuracy of the *i*-th tone’s phase estimation depends on the intensity of interference. Particularly, this accuracy depends on the relative magnitude ratio between $X_{q}\left(k_{i}\right),q\neq i$, and $X_{i}\left(k_{i}\right)$. From (28) and (29), we have

(30) $$\left\{\begin{array}{c} \frac{\left|X_{q}\left(k_{i}\right)\right|}{\left|X_{i}\left(k_{i}\right)\right|}=\frac{a_{q}}{a_{i}}\cdot \left|\frac{\sin\left(\left(k_{q}+\delta_{q}-k_{i}\right)\pi \right)/\sin(\left(k_{q}+\delta_{q}-k_{i}\right)\pi /N)}{\sin\left(\delta_{i}\pi \right)/\sin(\delta_{i}\pi /N)}\right|\\ \frac{\left|Y_{q}\left(k_{i}\right)\right|}{\left|Y_{i}\left(k_{i}\right)\right|}=\frac{a_{q}}{a_{i}}\cdot \frac{\sin^{2}\left(\left(k_{q}+\delta_{q}-k_{i}\right)\pi \right)/\sin^{2}\left(\left(k_{q}+\delta_{q}-k_{i}\right)\pi /N\right)}{\sin^{2}\left(\delta_{i}\pi \right)/\sin^{2}\left(\delta_{i}\pi /N\right)} \end{array}\right.$$

From (30), we have

(31) $$\frac{\left|Y_{q}\left(k_{i}\right)\right|/\left|Y_{i}\left(k_{i}\right)\right|}{\left|X_{q}\left(k_{i}\right)\right|/\left|X_{i}\left(k_{i}\right)\right|}=\left|\frac{\sin\left(\left(k_{q}+\delta_{q}-k_{i}\right)\pi \right)/\sin(\left(k_{q}+\delta_{q}-k_{i}\right)\pi /N)}{\sin\left(\delta_{i}\pi \right)/\sin(\delta_{i}\pi /N)}\right|$$

Please note that $0<\;|\delta_{i}\left|,\right|\delta_{q}|\;<0.5$, $|k_{q}-k_{i}|\;\geq 1$ and thus $|k_{q}+\delta_{q}-k_{i}|\;>0.5$. Since sin(δπ)/sin(δπ/N) is a monotonously descending even function, we have

(32) $$\left|\frac{\sin(\left(k_{q}+\delta_{q}-k_{i}\right)\pi )}{\sin(\left(k_{q}+\delta_{q}-k_{i}\right)\pi /N)}\right|<\left|\frac{\sin(\delta_{i}\pi )}{\sin(\delta_{i}\pi /N)}\right|$$

Combining (31) with (32), we have

(33) $$\left|\frac{Y_{q}\left(k_{i}\right)}{Y_{i}\left(k_{i}\right)}\right|<\left|\frac{X_{q}\left(k_{i}\right)}{X_{i}\left(k_{i}\right)}\right|,\;q\neq i.$$

Equation (33) shows that, for the fully-traversed DFT spectrum, the relative magnitude ratio between any other tone and the tone of interest is smaller than that of the conventional DFT. In other words, compared to the conventional DFT spectrum, the fully-traversed DFT magnitude spectrum does better in suppressing spectral leakage and interferences, thus yielding a higher accuracy of phase estimation.

### 3.3. Simplified Dataflow of Phase Estimation

From the aforementioned 4-step procedure listed in Section II, one can find that the fully-traversed DFT experiences *N* times of DFT operation. Therefore, to reduce computation complexity, this procedure needs to be simplified.

According to the linearity of DFT, the average of *N* sub-spectra is equivalent to the DFT result of the averaged data. Therefore, if we average the *N* sub-sequences ${\tilde{x}}_{0}∼{\tilde{x}}_{N-1}$ in (15) and (16), then one new sequence {y(n),n=0,1,⋯,*N* − 1} can be constructed as

(34) $$\begin{array}{cc} y(n) & =\frac{1}{N}\sum_{m=0}^{N-1}{\tilde{x}}_{m}\left(n\right)\\ & =\frac{N-n}{N}x\left(n\right)+\frac{n}{N}x\left(n-N\right),\;n=0,1,\ldots ,N-1 \end{array}$$

Accordingly, the DFT result *Y*(*k*) of *y*(*n*) is

(35) $$\begin{array}{c} Y(k)=\frac{1}{N}\sum_{n=0}^{N-1}\left[\frac{N-n}{N}x\left(n\right)+\frac{n}{N}x\left(n-N\right)\right]\;e^{-j\frac{2\pi }{N}nk},\\ k=0,1,\ldots ,N-1\; \end{array}$$

**Figure 1:**
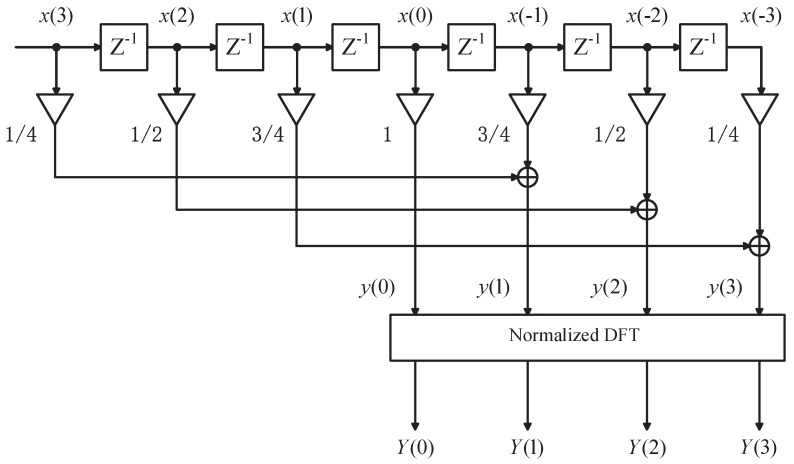
Simplified flow diagram of phase corrected DFT (*N* = 4).

Clearly, Equation (35) only involves one time of DFT operation, thus greatly reducing computation complexity. Accordingly, its simplified dataflow is illustrated in Figure 1 (take N=4 as an example), from which a low-complexity procedure of multi-tone phase estimation can be summarized as follows.

Firstly, weight the input (2N−1)-length data sequence [x(−N + 1),⋯,x(0),⋯,x(N − 1)] with one (2N − 1)-length triangular window [1/N,⋯,(N − 1)/N,1,(N − 1)/N,⋯,1/N];

Secondly, (*N* − 1) weighted data pairs (in each pair, the two data are spaced with *N* samples) are individually summed up to generate (*N* − 1) data y(1),⋯,y(N − 1), except the center sample x(0) due to y(0)=x(0);

Thirdly, implement normalized DFT on the data sequence [y(0),y(1),⋯,y(N − 1)] to provide the final spectral result Y = [Y(0),Y(1),⋯,Y(N − 1)].

Lastly, collect all the peak indices $k_{1},\ldots ,k_{Q}$ of Y(k). For each index $k_{q}$, directly taking the phase value of $Y(k_{q})$ provides its phase estimates ${\hat{\theta }}_{q}$.

## 4. Variance Analysis of Phase Estimation in Noisy Circumstances

### 4.1. CRLB for Conventional DFT Phase Estimator

Now we consider the phase estimation in noisy case, in which one random complex Gaussian process η(n) should be considered in (1), i.e.,

(36) $$\begin{array}{c} s\left(n\right)=x\left(n\right)+\eta \left(n\right)=a_{0}e^{j(\omega_{0}n+\theta_{0})}+\eta \left(n\right), \end{array}$$

where n=−N+1,⋯,N−1, $\omega_{0}=\beta \Delta \omega =\left(k_{0}+\delta \right)2\pi /N$, and η(n) is a complex Gaussian variable with mean zero and variance $\sigma^{2}$. As mentioned in (2), the conventional phase estimate is

(37) $$\begin{array}{cc} {\hat{\theta }}_{0} & =\varphi_{X}\left(k_{0}\right)-\left(1-1/N\right)\left(\beta -k_{0}\right)\pi \\ & =\varphi_{X}\left(k_{0}\right)-\left(1-1/N\right)\pi \delta . \end{array}$$

(37) indicates that, since the conventional DFT-based phase estimator relies on the frequency offset δ, the estimate error of frequency offset δ will propagate to the phase estimate. In fact, this dependency makes phase estimation obey a 3-parameter mathematical model parameterized with α = [$\omega_{0},\theta_{0},a_{0}{]}^{T}$. With regard to this model, previous studies [26,27] have derived a CRLB (Crammer-rao lower bound) for the variance of the phase estimate (consuming 2N−1 samples) as

(38) $$CRLB_{3}\left(\theta_{0}\right)=\frac{4N-3}{2N(2N-1)\rho },$$

where $\rho =a_{0}^{2}/2\sigma^{2}$ is the SNR (Signal to Noise Ratio). Constrained by CRLB${}_{3}$($\theta_{0}$) in (38), the error variance of phase estimate obtained by any conventional DFT-based estimator cannot exceed this bound. This has been especially claimed for algorithms in [1,2,5,6,7,8,9,27,28,29].

### 4.2. CRLB for the Proposed Phase Estimator

Different from conventional 3-parameter model-based phase estimators, mathematical model for the proposed corrected DFT-based phase estimator can be simplified. The reasons are as three aspects.

Firstly, (29) implies that the proposed phase detector only requires roughly searching the peak spectral bin and then taking the phases directly, i.e., independent of frequency offset δ.

Secondly, as previously mentioned, the proposed fully-traversed DFT spectrum equals the average of *N* sub DFT spectra. This actually reflects the following mechanism: averaging *N* sub vectors plays the role of compensating the angles of *N* sub DFT spectra with each other, which leads the synthesized phase to automatically fall at the ideal phase value, whether the frequency offset δ=0 or not.

Lastly, as previously mentioned, the proposed phase estimator can directly determine the ideal phase $\theta_{0}$, referring to the ‘instantaneous phase’ of the center sample among the 2N−1 input samples x(−N+1)∼x(N−1). Obviously, the position for the center sample is at n=0, which can be easily determined in advance. Thus, if we rewrite $x\left(n\right)=a_{0}e^{j(\omega_{0}n+\theta_{0})}$ as $a_{0}e^{j\varphi_{n}}$, then, for the position n=0, the entire term $\varphi_{n}$ equals the ideal phase $\theta_{0}$, which also indicates that estimation of frequency $\omega_{0}$ can be omitted.

For the above 3 reasons, the error variance of fully-traversed DFT phase estimator obeys a 2-parameter mathematical model with α = [$\theta_{0}$, $a_{0}$]${}^{T}$, in which the estimate of frequency offset δ is bypassed. Now we deduce the CRLB of this 2-parameter model using the classical parameter estimation theory [27].

Since η(n) is a Gaussian noise, the joint probability density function (pdf) of the (2N−1)-length observation sequence S = [$s_{-N+1},\cdots ,s_{0},\cdots ,s_{N-1}$]${}^{T}$ conditioned on the unknown vector α = [$\theta_{0},a_{0}$]${}^{T}$ is given by [26]

(39) $$\begin{array}{c} f\left(S|\alpha \right)={\left(\frac{1}{2\pi \sigma^{2}}\right)}^{\frac{2N-1}{2}}\exp\left(-\frac{1}{2\sigma^{2}}\sum_{n=-N+1}^{N-1}{\left(s_{n}-x_{n}\right)}^{2}\right) \end{array}$$

From (39), we can derive a 2 × 2 Fisher information matrix J whose entries are

(40) $$\begin{array}{cc} J_{ij} & =-E\left\{\frac{\partial \mathrm{In}f(S|\alpha )}{\partial \alpha_{i}\partial \alpha_{j}}\right\}\\ & =\frac{1}{\sigma^{2}}\sum_{n=-N+1}^{N-1}\frac{\partial x_{n}}{\partial \alpha_{i}}\cdot \frac{\partial x_{n}}{\partial \alpha_{j}},\;\;i,j=1,2. \end{array}$$

Since $x\left(n\right)=u\left(n\right)+jv\left(n\right)=a_{0}$cos$(\omega_{0}n+\theta_{0})+$j$a_{0}$sin$\left(\omega_{0}n+\theta_{0}\right)$, (40) can be further expressed as

(41) $$\begin{array}{c} J_{ij}=\frac{1}{\sigma^{2}}\sum_{n=-N+1}^{N-1}\left(\frac{\partial u_{n}}{\partial \alpha_{i}}\cdot \frac{\partial u_{n}}{\partial \alpha_{j}}+\frac{\partial v_{n}}{\partial \alpha_{i}}\cdot \frac{\partial v_{n}}{\partial \alpha_{j}}\right),i,j=1,2 \end{array}$$

with

(42) $$\frac{\partial u_{n}}{\partial \alpha_{1}}=\frac{\partial u_{n}}{\partial \theta_{0}}=-a_{0}\sin\left(\omega_{0}n+\theta_{0}\right),$$

(43) $$\frac{\partial u_{n}}{\partial \alpha_{2}}=\frac{\partial u_{n}}{\partial a_{0}}=\cos\left(\omega_{0}n+\theta_{0}\right),$$

(44) $$\frac{\partial v_{n}}{\partial \alpha_{1}}=\frac{\partial v_{n}}{\partial \theta_{0}}=a_{0}\cos\left(\omega_{0}n+\theta_{0}\right),$$

(45) $$\frac{\partial v_{n}}{\partial \alpha_{2}}=\frac{\partial v_{n}}{\partial a_{0}}=\sin\left(\omega_{0}n+\theta_{0}\right).$$

Substituting (42)∼(45) into (41) yields

(46) $$\begin{array}{cc} J_{11} & =\frac{{a_{0}}^{2}}{\sigma^{2}}\sum_{n=-N+1}^{N-1}\left(\sin^{2}\left(\omega_{0}n+\theta_{0}\right)+\cos^{2}\left(\omega_{0}n+\theta_{0}\right)\right)\\ & =\frac{{a_{0}}^{2}}{\sigma^{2}}\left(2N-1\right), \end{array}$$

(47) $$J_{12}=J_{21}=0,$$

(48) $$\begin{array}{cc} J_{22} & =\frac{1}{\sigma^{2}}\sum_{n=-N+1}^{N-1}\left(\sin^{2}\left(\omega_{0}n+\theta_{0}\right)+\cos^{2}\left(\omega_{0}n+\theta_{0}\right)\right)\\ & =\frac{1}{\sigma^{2}}\left(2N-1\right). \end{array}$$

Thus the 2 × 2 Fisher information matrix J takes the following diagonal form

(49) $$\begin{array}{c} J=\frac{1}{\sigma^{2}}\left[\begin{array}{cc} \left(2N-1\right)a_{0}^{2} & 0\\ 0 & 2N-1 \end{array}\right], \end{array}$$

and has the inverse as

(50) $$\begin{array}{c} J^{-1}=\sigma^{2}\left[\begin{array}{cc} \frac{1}{\left(2N-1\right)a_{0}^{2}} & 0\\ 0 & \frac{1}{\left(2N-1\right)a_{0}^{2}} \end{array}\right]. \end{array}$$

Thus, the CRLB for the error variance of the proposed phase estimator is

(51) $$CRLB_{2}\left(\theta_{0}\right)=\frac{\sigma^{2}}{\left(2N-1\right)a_{0}^{2}}=\frac{1}{2(2N-1)\rho }$$

Since the two CRLBs in (38) and (51) share a same sample length (2N−1), their ratio is

(52) $$\begin{array}{c} \frac{CRLB_{2}\left(\theta_{0}\right)}{CRLB_{3}\left(\theta_{0}\right)}=\frac{N}{\left(4N-3\right)}\approx \frac{1}{4}. \end{array}$$

Hence, the CRLB for 2-parameter joint estimation is only 25% of that for 3-parameter joint estimation.

### 4.3. Numerical Results

**Figure 2:**
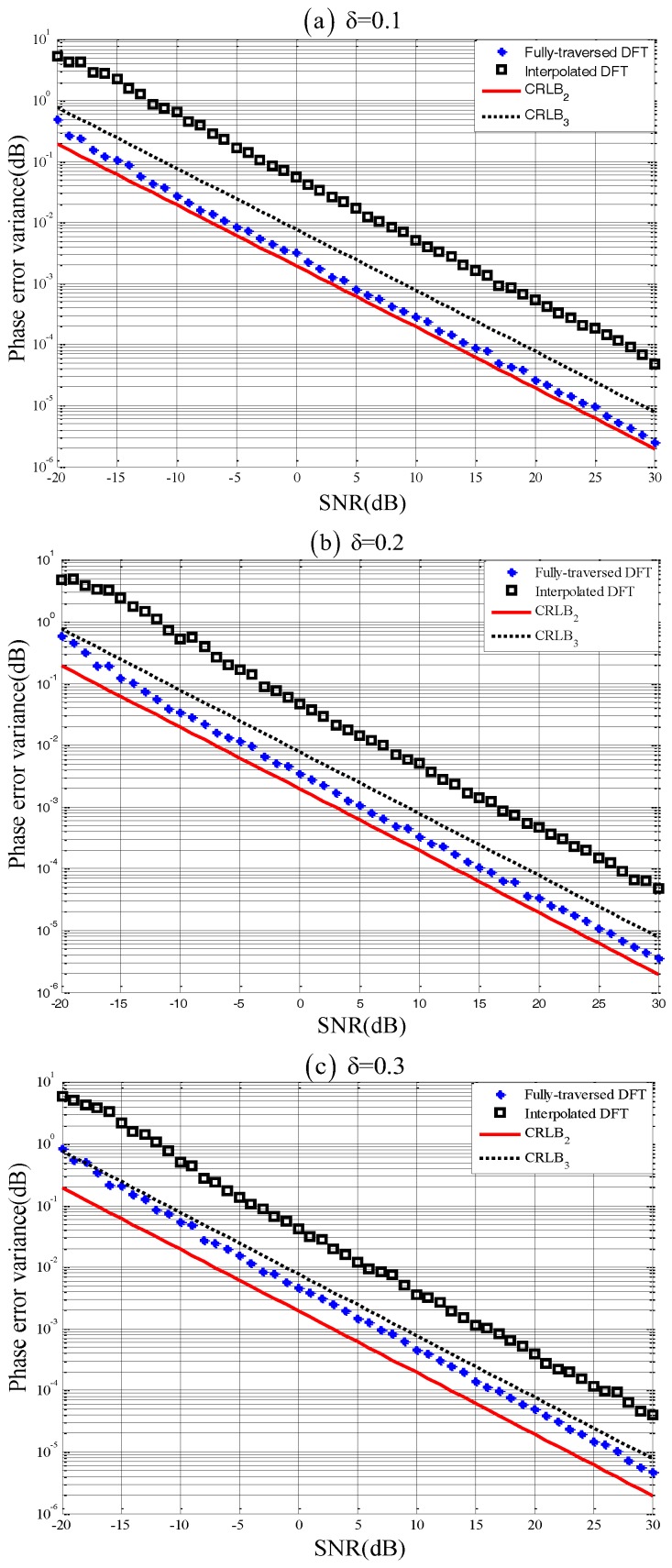
Error variances of the phase estimator with (**a**) δ=0.1, (**b**) δ=0.2 and (**c**) δ=0.3.

To further verify the superiority of the proposed phase estimator, simulations performed under various noisy conditions and different spectral orders are presented. The phase error variance of the proposed estimator was also compared with that of conventional DFT-based estimator (we choose the ratio method based on interpolated DFT [2]). Assume $k_{0}=3$, N=128 and $\theta_{0}=60^{\circ }$. Then, the specific signal based on (36) is

(53) $$\begin{array}{c} s\left(n\right)=a_{0}e^{j\left[\left(3+\delta \right)\times \frac{2\pi }{N}n+\frac{\pi }{3}\right]}+\eta \left(n\right), \end{array}$$

where the frequency offset δ is specified with 3 values: 0.1,0.2,0.3. Figure 2a–c gives the error variance curves versus SNRs for these 3 frequency offsets, respectively. For each SNR and δ case, 500 Monte Carlo trials were conducted.

As can be seen in each figure, the majority of phase corrected DFT’s error variance curve (marked in ‘∗’) lies below CRLB${}_{3}$, proving that the proposed phase estimator is independent of the conventional 3-parameter joint estimation model. Furthermore, the proposed estimator’s error variance curve is bounded by CRLB${}_{2}$ curve, verifying the correctness of (51). Figure 2 also demonstrates that the error variances of the conventional interpolated DFT estimator are nearly one order of magnitude higher than that of the proposed estimator.

## 5. Applying Fully-Traversed DFT in Phase-Coded SSVEP-BCI

Recently, BCIs have become a very hot topic in neural engineering. A BCI detects an user’s ongoing brain activities and translates them into meaningful messages, which helps patients with severe motor disabilities to express their messages to external world [30]. In particular, BCI based steady-state visual evoked potential (SSVEP) has received much attention in bioengineering research due to its satisfactory performance [31]. To increase the number of recognizable targets, the phase-coded SSVEP-BCIs use phase information to encode subject’s visual intention. In this system, the phase-tagged visual stimuli are characterized with flashing at one frequency but different phases, resulting in that subjects’ SSVEPs also differ in phase features. Therefore, through extracting the phase information of SSVEP potentials, a computer is able to distinguish which flicker the subject desires to select.

### 5.1. Experiment Paradigm

In general, SSVEP is always elicited after some latency time *L* (or labeled as lag phase ‘$\theta_{L}$’), which actually corresponds to a phase difference between flicker’s phase ‘$\theta_{F}$’ and SSVEP’s phase ‘$\theta_{S}$’. Hence, the relationship between the phase difference ‘$\theta_{L}$’ and latency *L* is described by

(54) $$\theta_{S}=\theta_{F}+\theta_{L}$$

(55) $$\theta_{L}=-\left(L\times 360\times f_{s}-q\times 360\right)$$

where $f_{s}$, *q* denote the stimulus frequency and the integer cycles, respectively. Under normal condition, *L* is stable in a short period of time but differs in inter-subject such that $\theta_{L}$ cannot be calculated in advance [21,23,24]. From (54), it can be found that the flicker’s phase is usually not equal to SSVEP’s phase (or $\theta_{F}\neq \theta_{S}$) due to $\theta_{L}$, which means that we cannot directly use $\theta_{s}$ to identify which flicker the subject desires to select if $\theta_{L}$ is unknown. As a result, an additional measure of phase calibration is necessary for phase-coded SSVEP-BCI to calibrate this error $\theta_{L}$ in the detection algorithm.

Hence, we build up a BCI system which uses a half-field phase-tagged stimulus to evoke the SSVEP with two different frequency components $f_{1}$, $f_{2}$ at the same time. This system does not adopt any phase calibration since it is able to identify the flicker by introducing the phase difference instead of the phase under the assumption $f_{1}\neq f_{2}$ (this frequency distinction makes a subject more sensitive to flickering stimuli than to those with the same frequency), i.e.,

(56) $$\begin{array}{c} \theta_{S}\left(f_{1}\right)-\theta_{S}\left(f_{2}\right)=\theta_{F}\left(f_{1}\right)-\theta_{F}\left(f_{2}\right)+\theta_{L}\left(f_{1}\right)-\theta_{L}\left(f_{2}\right) \end{array}$$

If $f_{1}\approx f_{2}$, then $\theta_{L}\left(f_{1}\right)\approx \theta_{L}\left(f_{2}\right)$. Therefore, the difference between $\theta_{S}\left(f_{1}\right)$ and $\theta_{S}\left(f_{2}\right)$ approximately equals the difference between $\theta_{F}\left(f_{1}\right)$ and $\theta_{F}\left(f_{2}\right)$. Hence, the lag phase difference can be removed.

**Figure 3:**
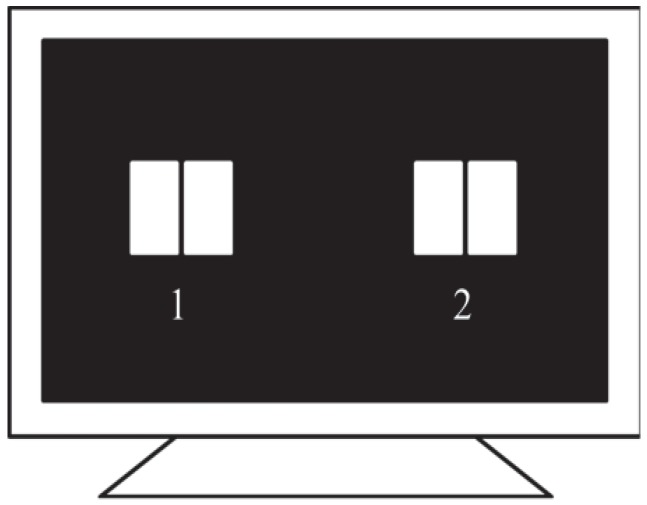
Visual stimulator presenting two half-field phase-tagged stimuli. (Left field of Flicker 1: 10.9 Hz and 0 deg, Right field of Flicker 1: 12 Hz and 0 deg, Left field of Flicker 2: 10.9 Hz and 0 deg, Right field of Flicker 2: 12 Hz and 180 deg).

In our phase-coded SSVEP-BCI system, a visual stimulator (ViewSonic, 22 inch, 120 Hz refresh rate, 1680×1050 screen resolution) presenting two phase-tagged flickers (with the size 12 cm × 8 cm each) was used to evoke subjects’ SSVEPs (Figure 3 and Table 1).

It should be noted that, in our SSVEP-BCI illustrated in Figure 3, the selected flickering frequencies $f_{1}$ and $f_{2}$ should be as close as possible (this helps to remove the possible jump change of multiple of $360^{\circ }$ for the lag phase difference between $\theta_{L}\left(f_{1}\right)$ and $\theta_{L}\left(f_{2}\right)$, which was solved in [17] by means of a exhaustive search procedure based on least-square fitting). Otherwise, their lag phase difference $\left(\theta_{L}\left(f_{1}\right)-\theta_{L}\left(f_{2}\right)\right)$ would not be removed. However, due to the fact that all the stimulus frequencies in our SSVEP-BCI are acquired by integer dividing a fixed LCD display refresh frequency $F_{r}$ = 120 Hz (see [32]), we can only obtain a limited number of flikering frequencies (they are 120/7 Hz, 120/8 Hz, 120/9 Hz, 120/10 Hz, 120/11 Hz) falling at the visual sensitive region (10 Hz, 20 Hz). Therefore, among these candidate flickering frequencies, the frequency pair with the minimum interval is ($f_{1},f_{2}$) = (120/11 Hz, 120/10 Hz) = (10.9 Hz, 12 Hz). Obviously, in this case, the lag phase difference between $\theta_{L}\left(f_{1}\right)$ and $\theta_{L}\left(f_{2}\right)$ will not be removed as $f_{1}$ and $f_{2}$ are not close enough. Hence, this phase difference should be taken into account in order to achieve an accurate result. In practice, it can be roughly estimated according to their empirical results of the apparent latency of SSVEPs [24]. In our experiment, $\left(\theta_{L}\left(f_{1}\right)-\theta_{L}\left(f_{2}\right)\right)$ cam be roughly estimated as $36^{\circ }$.

Different from the well-known CCA (Canonical Correlation Analysis) method, which also uses phase information to enhance the classification accuracy of SSVEPs, our proposed scheme consumes lower hardware cost. As [17] pointed out, the CCA method needs multiple stimulus frequencies (6 frequencies were adopted) to remove the possible jump change of multiple of $360^{\circ }$ for the lag phase difference. In contrast, our proposed scheme only employs 2 frequencies, thereby lowering the hard cost.

Three subjects (S1∼S3, two males and one female) were seated on a comfortable chair before the visual stimulator in an illuminated room. The subjects’ EEG signals were recorded by a g.USBamp EEG amplifier from 13 electrodes (PO${}_{3}$, PO${}_{5}$, PO${}_{7}$, POZ, PO${}_{4}$, PO${}_{6}$, PO${}_{8}$, P1, PZ, P2, O1, Oz, and O${}_{2}$). Specify the sampling rate $F_{s}=600$ samples/s. This experiment consisted of 5 runs containing 10 trials each. Each trial lasted for 8 s. Subjects were instructed to focus on one of flickers according to the following paradigm: From 0 to 2 s a cue appeared indicating which flashing flicker was required to focus on; From 2 s to 8 s the subjects gazed at the specified flicker; Then the next trial started. The order of gazed-flickers was ‘1212121212’ in each run. Thus this dataset had 25 trials for each flicker. The whole experiment lasted about 30 minutes. During this experiment, the subjects’ EEG signals were recorded for offline analysis later.

### 5.2. Procedure of SSVEP Phase Extraction

We collected a total amount of 50 trials for each subject. Basically, the procedure of SSVEP phase extraction would contain the following steps:

**step** **1.**  Apply both conventional DFT and phase corrected DFT to extract the phase values $\theta_{S}$($f_{1}$) and $\theta_{S}\left(f_{2}\right)$, respectively;
**step** **2.**  Substitute $\theta_{S}\left(f_{1}\right)-\theta_{S}\left(f_{2}\right)$ and the estimate $\left(\theta_{L}\left(f_{1}\right)-\theta_{L}\left(f_{2}\right)\right)$ = $36^{\circ }$ into (56) to estimate the difference $\Delta \hat{\theta }$ = $\theta_{F}\left(f_{1}\right)-\theta_{F}\left(f_{2}\right)$;
**step** **3.**  Use the estimate $\Delta \hat{\theta }$ to identify the gazed-flicker. If $\Delta \hat{\theta }$ is close to 0 (or 180) deg, then the gazed-flicker is judged as flicker 1 (or 2).

The judgement involved in step 3 can actually be extended to distinguish *C* targets (C≥2) by finding the maximum among *C* decision variables $R_{k}$ as

(57) $$R_{k}=\cos\left(\Delta \hat{\theta }-\varphi_{k}\right),\;k=1,\ldots ,C$$

where $\varphi_{k}$ refers to the ideal phase difference $\left(\theta_{F}\left(f_{1}\right)-\theta_{F}\left(f_{2}\right)\right)$, i.e., the ideal clustering center of pattern recognition. For the above 2-category recognition problem, we have $\varphi_{1}=0^{\circ }$, $\varphi_{2}=180^{\circ }$. From (57), it can be found if the detected phase difference $\Delta \hat{\theta }\to \varphi_{k}$, then $R_{k}\to 1$. In other words, if $R_{1}$ is close to 1, the gazed-flicker is judged as flicker 1 and vice versa. Furthermore, (57) also allows to assume more clustering centers. As will be elaborated, introducing more assumed clustering centers helps to evaluate the performance of phase estimator.

### 5.3. Result of Offline Analysis

The classification rates of this 2-class experiment are listed in Table 2.

It can be found that the proposed phase estimator can achieve a higher classification rate than conventional DFT-based estimator does, which is entirely over 10%.

Table 3 lists not only these two estimators’ detected phase values ($\theta_{s}$(12), $\theta_{s}$(10.9)) but also 4 columns of phase decision values $R_{k}\left(j\right),k=1,2,3,4$, calculated by (57). Here decision variable $R_{k}\left(j\right)$ corresponds to the *k*th assumed clustering center (4 assumed phase clustering centers are $0^{\circ },180^{\circ },90^{\circ },270^{\circ }$, respectively) while the subject actually gazes at the flicker *j*. Therefore, for any flicker index j(j=1,2), $R_{j}\left(j\right)$ (marked with shadow in Table 3) is close to 1 and tends to be the maximum among $R_{k}\left(j\right),k=1,2,3,4$, if the accuracy of selected phase estimator is high enough. The data for all trials involved in Table 3 are recorded within 4 s.

In Table 3, generally speaking, the detected phases $\theta_{S}\left(10.9\right)$ by the proposed phase estimator are closer to the ideal phase ‘$0^{\circ }$’ than that of conventional DFT estimator. This result can be explained as follows: Since the sampling rate $F_{s}=600$ samples/s and the window length equals 4s, it follows that N=2400 samples are recorded and thus DFT frequency resolution $\Delta f=F_{s}/N=0.25$ Hz. Hence, the stimulus frequency $f_{1}=10.9$ Hz can also be written as $f_{1}=43.6\Delta f=\left(44-0.4\right)\Delta f$, i.e., the frequency offset δ equals nonzero value −0.4, indicating that severe spectral leakage causing large phase measurement error will arise in the DFT spectrum. In contrast to this, for our proposed phase estimator, due to the property of ‘direct phase extraction’, the phase measurement error caused by frequency offset is much smaller than that of the conventional DFT case, as Table 3 lists.

Different from the case of $\theta_{S}\left(10.9\right)$, both estimators’ detected phases $\theta_{S}\left(12\right)$ are uniformly close to the ideal phase $0^{\circ }$ (or $180^{\circ }$) (Even both mean and standard deviation are similar). This is because $f_{2}=12\;$Hz =48Δf, i.e., the frequency offset δ=0, resulting in that spectral leakage will not appear in both conventional DFT spectra and corrected DFT spectra.

Hence, since SSVEP phase extraction in this experiment is based on the phase difference between $\theta_{S}\left(10.9\right)$ and $\theta_{S}\left(12\right)$ rather than either of them, the conventional DFT phase estimator is more likely to cause large errors than the proposed phase estimator. From Table 3, one can notice that, for the conventional DFT phase estimator, the phase differences between $\theta_{S}\left(10.9\right)$ and $\theta_{S}\left(12\right)$ are far away from 36 deg (see the results in 3-rd, 7-th and 11-th row, respectively). Accordingly, the expected maximum decision values $R_{1}\left(1\right)$ or $R_{2}\left(2\right)$ detected by conventional DFT estimator are entirely smaller than that detected by the proposed estimator, as Table 3 lists. This reflects the fact that the conventional DFT is not so good as the proposed fully-traversed DFT in extracting the phase information of SSVEP.

To further evaluate the performance of the fully-traversed DFT in this experiment, as previously mentioned, we assume that there were 4 flickers with stimulus phases $0^{\circ },180^{\circ },90^{\circ },270^{\circ }$ on screen. Hence, the gazed-flicker was identified by finding the maximum among $R_{1}$, $R_{2}$, $R_{3}$, $R_{4}$. Moreover, the average classification rate for these 4 assumed flickers are also listed in Table 4. In this simulated 4-category experiment, compared to the 2-category case in Table 2, the classification rate of the fully-traversed DFT decreases but still over 80%. In contrast, the classification rate of conventional DFT is only about 37%, as Table 4 lists. The underlying reason lies in that conventional DFT cannot accurately extract the phase information at 10.9 Hz due to spectral leakage, while the proposed phase corrected DFT still works well for its property of ‘direct phase extraction’.

In summary, fully-traversed DFT is well suitable for the phase-coded SSVEP-BCI, especially in our proposed duel-frequency stimulus-based system which uses phase difference to recognize the gazed flick. Due to the fact that fully-traversed DFT does well in extracting the phase information at any frequency offset, the proposed phase estimator can achieve a higher classification rate. More importantly, as listed in Table 3, we find that the corresponding phase’s standard deviation of the proposed estimator is smaller than that of conventional DFT estimator, although the frequency resolution of fully-traversed DFT is less than DFT. The reason is actually mentioned in Section 2, i.e., the mechanism that fully-traversed DFT is the average of *N* DFT sub-spectra (see formula (18)) also helps to reduce the averaged noise’s power.

Based on our preliminary results, we believe that fully-traversed DFT can be applied in the online system. Although the simulated 4-category experiment results show that the corresponding average classification rate is only 80%, the classification rate is surely to be further enhanced once the estimate of the lag phase difference is more accurate. In this experiment, we only roughly estimate it as 36 deg. In fact, we can use another two very close frequencies (for example, we can replace LCD stimuli with LED stimuli) such that the lag phase difference will be close to zero and thus we do not need to estimate it. Moreover, it was also clearly found that the phase variance of fully-traversed DFT estimator does not get worse than conventional DFT under our experiment condition.

## 6. Conclusions

A novel phase estimator based on corrected-phase DFT was proposed in this paper. Due to considering all possible truncated sequences containing the center sample, spectral leakage in corrected-phase DFT is greatly reduced and thus the instantaneous phase information of the center sample can be directly extracted. In addition, we have also proved that the process of corrected-phase DFT is equivalent to a streamline dataflow, and thus the proposed phase estimator has a relatively low computational complexity. Furthermore, the phase error variance of the proposed phase estimator follows a 2-parameterized mathematical model and thus it has a higher accuracy than the conventional DFT-based phase estimators in noisy circumstances.

We also applied the proposed phase estimators to phase-coded SSVEP-BCIs. In particular, compared to the conventional DFT-based estimators, our offline experiment results demonstrate that the fully-traversed DFT does better in extracting the phase information of phase-coded SSVEP-BCI. Moreover, the proposed phase estimator imposes on restrictions on the relationship between the sampling rates and the stimulus frequencies (i.e., non-synchronous sampling is allowed), it is of wider applications in phase-coded SSVEP BCIs than the existing estimators. Our future work is to improve our system design and apply fully-traversed DFT in the practical SSVEP-BCI online system.

## Figures and Tables

**Table 1:** The parameters of two phase-tagged half-field flickers.

	Left-Field		Right-Field	
	Freq. (Hz)	Phase (deg)	Freq. (Hz)	Phase (deg)
Flicker 1	10.9	0	12	0
Flicker 2	10.9	0	12	180

**Table 2:** The classification rate of each subject under different window length.

Subject	Method	Electrode	Window Length (sec)				Average
			3	4	5	6	
S1	Proposed	POZ	0.94	1.00	1.00	0.98	0.98
	DFT	PO7	0.92	0.88	0.78	0.78	0.84
S2	Proposed	O2	0.98	1.00	1.00	1.00	1.00
	DFT	P2	0.92	0.88	0.84	0.64	0.82
S3	Proposed	P1	0.80	0.96	0.92	0.94	0.91
	DFT	PZ	0.92	0.94	0.88	0.74	0.87
Average	Proposed	–	0.91	0.99	0.97	0.97	0.96
	DFT	–	0.92	0.90	0.83	0.73	0.84

**Table 3:** The phase and phase feature $R_{j}$ of each subject (mean ± S.D.)

Subject	Method	Flicker j	$\theta_{S}$(12) (deg)	$\theta_{S}$(10.9) (deg)	$R_{1}$(j)	$R_{2}$(j)	$R_{3}$(j)	$R_{4}$(j)
S1	Proposed	1	322.74 ± 29.3	6.84 ± 31.4	0.985 ± 0.03	0.133 ± 0.11	0.741 ± 0.1	0.651 ± 0.13
		2	126.52 ± 23.1	2.25 ± 27.2	0.253 ± 0.16	0.954 ± 0.05	0.56 ± 0.18	0.789 ± 0.18
	DFT	1	329.22 ± 39.3	57.09 ± 38.0	0.857 ± 0.17	0.416 ± 0.26	0.896 ± 0.1	0.378 ± 0.22
		2	151.46 ± 31.9	49.97 ± 34.1	0.422 ± 0.22	0.865 ± 0.16	0.445 ± 0.23	0.848 ± 0.19
S2	Proposed	1	334.22 ± 36.1	23.10 ± 35.5	0.983 ± 0.03	0.132 ± 0.12	0.771 ± 0.09	0.62 ± 0.12
		2	146.96 ± 31.0	17.47 ± 35.7	0.193 ± 0.13	0.972 ± 0.04	0.601 ± 0.16	0.774 ± 0.12
	DFT	1	332.91 ± 32.2	72.68 ± 37.7	0.833 ± 0.11	0.511 ± 0.19	0.95 ± 0.06	0.25 ± 0.18
		2	148.24 ± 29.4	50.71 ± 28.8	0.421 ± 0.19	0.881 ± 0.12	0.362 ± 0.16	0.916 ± 0.07
S3	Proposed	1	15.04 ± 42.4	34.66 ± 43.2	0.899 ± 0.11	0.347 ± 0.25	0.538 ± 0.31	0.75 ± 0.24
		2	198.30 ± 26.0	24.81 ± 35.5	0.339 ± 0.21	0.913 ± 0.09	0.822 ± 0.19	0.471 ± 0.27
	DFT	1	9.71 ± 39.0	73.81 ± 36.8	0.899 ± 0.13	0.349 ± 0.24	0.794 ± 0.22	0.508 ± 0.27
		2	198.94 ± 24.5	81.04 ± 38.3	0.323 ± 0.26	0.892 ± 0.19	0.522 ± 0.22	0.814 ± 0.15
Average	Proposed	1	–	–	0.956 ± 0.06	0.204 ± 0.16	0.683 ± 0.17	0.674 ± 0.17
		2	–	–	0.262 ± 0.17	0.946 ± 0.06	0.661 ± 0.18	0.678 ± 0.19
	DFT	1	–	–	0.863 ± 0.14	0.425 ± 0.23	0.88 ± 0.13	0.379 ± 0.22
		2	–	–	0.389 ± 0.22	0.879 ± 0.16	0.443 ± 0.2	0.859 ± 0.14

**Table 4:** The classification rate of each subject under different window length (Simulated 4-classes experiment).

Subject	Method	Electrode	Window Length (sec)				Average
			3	4	5	6	
S1	Proposed	POZ	0.80	0.90	0.90	0.84	0.86
	DFT	PO7	0.67	0.43	0.18	0.18	0.37
S2	Proposed	O2	0.92	0.90	0.98	0.90	0.93
	DFT	P2	0.54	0.28	0.16	0.06	0.26
S3	Proposed	P1	0.54	0.58	0.68	0.74	0.64
	DFT	PZ	0.56	0.62	0.46	0.30	0.49
Average	Proposed	–	0.75	0.79	0.85	0.83	0.81
	DFT	–	0.59	0.44	0.27	0.18	0.37
